# Up North and Down South: Regional Differences in Pain, Religious Coping, and Negative Affect in Osteoarthritis

**DOI:** 10.1093/geroni/igaa057.1223

**Published:** 2020-12-16

**Authors:** Katherine Cheesman, Patricia Parmelee, Dylan Smith

**Affiliations:** 1 The University of Alabama, Tuscaloosa, Alabama, United States; 2 University of Alabama, Tuscaloosa, Alabama, United States; 3 Stony Brook University, Stony Brook, New York, New York, United States

## Abstract

Objective: This research examines regional differences (Northern vs. Southern) in pain, religious coping, and negative affect among African Americans (AA) and non-Hispanic Whites (NHW) over the age of 50 with physician-confirmed knee osteoarthritis (OA). Methods: As part of a larger study of racial/ethnic differences in everyday quality of life with OA, 116 persons were recruited from sites in Alabama (n = 64) and New York (n = 52). Participants completed global measures of pain (PGC Pain Scale) and religious coping (Brief RCOPE); daily variability in pain, coping, and affect was assessed using a daily diary methodology consisting of 4 daily phone calls over 7 days. Site comparisons were conducted using one-way multivariate analysis of covariance (MANCOVA) with covariates of race, sex, education, and marital status. Results: There was a significant multivariate effect of site on pain, religious coping, and affect, F(5, 104) = 3.846, p = .003, Wilk’s Λ = .844, partial η2 = .156. Follow-up univariate tests and mean examinations revealed that Southerners reported statistically more daily pain (M = 2.023, SD = .89), religious coping (M = .618, SD = .427), and negative affect (M = 6.556, SD = 2.661) than Northerners (M = 1.810, SD = .719; M = .386, SD = .417; M = 5.865, SD = 1.446). Implications: Results contribute to a growing understanding of how individuals use their religious beliefs to cope with daily pain. (Supported by R01-AG041655 D. Smith and P. Parmelee, PIs.)

